# POLICY SERIES: EXPLORING DISPARITIES IN LONG-TERM SERVICES AND SUPPORTS FOR LGBTQIA+ OLDER ADULTS

**DOI:** 10.1093/geroni/igae098.1611

**Published:** 2024-12-31

**Authors:** Jason Flatt, Tetyana Shippee, Mark Brennan-Ing

**Affiliations:** Chair; Co-Chair; Discussant

## Abstract

Lesbian, gay, bisexual, transgender, queer, intersex, and/or asexual (LGBTQIA+) older adults are a growing population that has been mostly ignored in research and development of long-term services and supports (LTSS). Due to fears of prejudice and discrimination, institutional policies, and state and federal laws, many LGBTQIA+ older adults may be reluctant to use LTSS. In this symposium, we explore micro-, meso-, and macro-level influences that may impact inequities in LTSS access and use. First, Dr. Jason Flatt will focus on micro- and macro-level factors from a national study with LGBTQIA+ older adults that explores how past experiences of discrimination may result in barriers for aging-related care and LTSS planning. The second presentation by Dr. Rajean Moone will examine micro- and meso-level factors via experiences of 354 LGBTQIA+ older adults from Minnesota on needs for LTSS and training of providers. Next, Dr. Tetyana Shippee will share meso- and macro-level findings via an analysis of LGBTQIA+ affirmative LTSS policies across 166 nursing homes and 267 assisted living facilities in Minnesota. Finally, Dr. Carey Candrian will provide meso- and macro-level perspectives in terms of a training program and lessons learned from a cultural change project with over 800 staff from Christian Living Communities on inclusion of LGBTQIA+ in LTSS. Dr. Mark Brennan-Ing an expert in LGBTQIA+ aging research will facilitate a conversation about the conclusions from each speaker’s presentation and place them in the context of efforts needed at micro-, meso- and macro-levels to ensure inclusive LTSS practices and policies for LGBTQIA+ elders.

